# 液相色谱-串联质谱法测定土壤、沉积物和水中3种新型除草剂残留

**DOI:** 10.3724/SP.J.1123.2023.07006

**Published:** 2024-03-08

**Authors:** Hongmei HE, Lingying XU, Changpeng ZHANG, Nan FANG, Jinhua JIANG, Xiangyun WANG, Jianzhong YU, Xueping ZHAO

**Affiliations:** 省部共建农产品质量安全危害因子与风险防控国家重点实验室, 农业农村部农药残留检测重点实验室, 浙江省农业科学院农产品质量安全与营养研究所, 浙江 杭州 310021; State Key Laboratory for Managing Biotic and Chemical Threats to the Quality and Safety of Agro-products, Key Laboratory for Pesticide Residue Detection of Ministry of Agriculture and Rural Affairs, Institute of Agro-product Safety and Nutrition, Zhejiang Academy of Agricultural Sciences, Hangzhou 310021, China

**Keywords:** 固相萃取, 液相色谱-串联质谱, 除草剂, 土壤, 沉积物, 水, solid-phase extraction (SPE), liquid chromatography-tandem mass spectrometry (LC-MS/MS), herbicides, soil, sediment, water

## Abstract

除草剂在杂草和有害植物防控上发挥着重要的作用,但其有效利用率低,大量除草剂进入环境中,对生态环境和人类健康构成了潜在威胁,因此建立环境样品中除草剂的残留分析方法尤为重要。该文采用电喷雾正离子源模式,建立了液相色谱-串联质谱法(LC-MS/MS)测定土壤、沉积物和水中异噁唑草酮、吡唑草胺和苯嘧磺草胺残留量的方法。土壤和沉积物样品经乙腈振荡提取、盐析后经C_18_固相萃取小柱净化,水样过滤后经C_18_固相萃取小柱净化;再用LC-MS/MS测定样品中异噁唑草酮、吡唑草胺和苯嘧磺草胺的残留量。实验优化了仪器检测和前处理条件,考察了方法的线性关系、基质效应、检出限和定量限,并选取4种土壤、2种沉积物和水样进行了方法验证。在0.0005~0.02 mg/L范围内,异噁唑草酮、吡唑草胺、苯嘧磺草胺的线性关系均良好,*r*≥0.9961。3种除草剂在土壤、沉积物和水中的基质效应为-10.1%~16.5%。异噁唑草酮、吡唑草胺和苯嘧磺草胺的检出限分别为0.05、0.02、0.01 μg/kg,定量限分别为0.2、0.05、0.05 μg/kg。异噁唑草酮、吡唑草胺和苯嘧磺草胺在土壤、沉积物和水样中3个水平(0.005、0.1、2.0 mg/kg)下的加标回收率分别为77.2%~101.9%、77.9%~105.1%、80.8%~107.1%;相对标准偏差(RSD)分别为1.4%~12.8%、1.2%~7.7%、1.5%~11.5%。结果表明:本方法操作简单,方法稳定,定量准确,实用性强,可用于土壤、沉积物和水中异噁唑草酮、吡唑草胺和苯嘧磺草胺残留量的检测。

## Abstract (en)

Herbicides play an important role in preventing and controlling weeds and harmful plants and are increasingly used in agriculture, forestry, landscaping, and other fields. However, the effective utilization rate of herbicides is only 20%-30%, and most herbicides enter the atmosphere, soil, sediment, and water environments through drift, leaching, and runoff after field application. Herbicide residues in the environment pose potential risks to ecological safety and human health. Therefore, establishing analytical methods to determine herbicide residues in environmental samples is of great importance. In this study, an analytical method based on liquid chromatography-tandem mass spectrometry (LC-MS/MS) in positive electrospray ionization mode (ESI^+^) was developed for the determination of isoxaflutole, metazachlor, and saflufenacil residues in soil, sediment, and water. The instrumental detection parameters, including electrospray ionization mode, mobile phase, and chromatographic column, were optimized. The mobile phases were methanol (A) and 0.1% formic acid aqueous solution (B). Gradient elution was performed as follows: 0-1.0 min, 60%A; 1.0-2.0 min, 60%A-90%A; 2.0-3.0 min, 90%A; 3.0-4.0 min, 90%A-60%A; 4.0-5.0 min, 60%A. The samples were salted after extraction with acetonitrile and cleaned using a C_18_ solid-phase extraction column. Different solid-phase extraction columns and leaching conditions were investigated during sample pretreatment. Working curves in the neat solvent and matrix were constructed by plotting the measured peak areas as a function of the concentrations of the analytes in the neat solvent and matrix. Good linearities were found for isoxaflutole, metazachlor, and saflufenacil in the solvent and matrix-matched standards in the range of 0.0005-0.02 mg/L, with *r*≥0.9961. The matrix effects of the three herbicides in soil, sediment, and water ranged from -10.1% to 16.5%. The limits of detection (LODs, *S/N*=3) for isoxaflutole, metazachlor, and saflufenacil were 0.05, 0.01, and 0.02 μg/kg, respectively. The limits of quantification (LOQs, *S/N*=10) for isoxaflutole, metazachlor, and saflufenacil were 0.2, 0.05, and 0.05 μg/kg, respectively. The herbicides were applied to soil, sediment, and water at spiked levels of 0.005, 0.1, and 2.0 mg/kg, respectively. The average recoveries for isoxaflutole, metazachlor, and saflufenacil in soil, sediment, and water were in the ranges of 77.2%-101.9%, 77.9%-105.1%, and 80.8%-107.1%, respectively. The RSDs for isoxaflutole, metazachlor, and saflufenacil were in the ranges of 1.4%-12.8%, 1.2%-7.7%, and 1.5%-11.5%, respectively. The established method was used to analyze actual samples collected from four different sites in Zhejiang Province (Xiaoshan, Taizhou, Dongyang, and Yuhang) and one site in Heilongjiang (Jiamusi). The proposed method is simple, rapid, accurate, stable, and highly practical. It can be used to detect isoxaflutole, metazachlor, and saflufenacil residues in soil, sediment, and water and provides a reference for monitoring the residual pollution and environmental behavior of herbicides.

除草剂主要用于农业和非农业杂草和有害植物的防控,是保障农业生产的重要投入品。除草剂应用广,种类多,包括有机磷类(如草甘膦)、酰胺类(如乙草胺)、磺酰脲类(如烟嘧磺隆)、嘧啶水杨酸类(如双草醚)、三嗪类(如莠去津)、联吡啶类(如百草枯)、芳氧苯氧丙酸类(如氰氟草酯)等^[1]^。但实际生产中除草剂的有效利用率仅为20%~30%,大部分进入环境中^[2]^,多种除草剂对非靶标生物和人体存在生殖毒性与遗传毒性^[1]^,对生态环境和人类健康构成潜在威胁^[2⇓-4]^。因此,建立环境样品中除草剂的残留分析方法尤为重要。

随着除草剂的广泛应用,很多产品出现了药害、残留污染、杂草抗药性以及毒性等问题。因此,新型除草剂的应用备受关注。近年来,异噁唑草酮(isoxaflutole, ISO)、吡唑草胺(metazachlor, MET)和苯嘧磺草胺(saflufenacil, SAF)等新型除草剂产品在我国获得登记。截至2023年7月,ISO、SAF和MET制剂产品分别为33个、7个和1个^[5]^。ISO是一种由拜耳公司研发的对羟基苯基丙酮酸双氧化酶(HPPD)抑制剂类选择性除草剂^[6,7]^,主要防治玉米田一年生杂草。MET属于氯乙酰苯胺类除草剂,通过抑制长链脂肪酸的形成防除油料作物田中一年生草本和阔叶杂草^[4]^。SAF是一种由巴斯夫公司开发的嘧啶类非选择性除草剂,能有效防除多种阔叶杂草^[8,9]^。

目前,国内外关于这3种新型除草剂的相关报道有合成^[10]^、分解^[4]^、药效^[7,8,11⇓⇓⇓⇓⇓⇓-18]^、生态风险评估^[19]^、环境行为特性^[20⇓-22]^、残留分析方法^[5,6,23⇓⇓⇓⇓⇓⇓⇓⇓⇓⇓-34]^等。残留分析方法涉及的基质有谷物^[5,6,28,29]^、畜禽^[27,30]^、水产^[24,26,33]^、加工食品^[23,25,34]^、土壤^[5,20⇓-22,32,34]^、沉积物^[21,33]^和水^[21,33]^等。检测方法为液相色谱法(HPLC)^[20⇓-22,31]^、液相色谱-串联质谱法(LC-MS/MS)^[5,6,23⇓⇓⇓⇓⇓⇓-30,33,34]^和气相色谱法(GC)^[32]^。涉及环境样品的净化方法有固相萃取法^[20,21,30,33]^、液-液分配法^[22]^、QuEChERS方法^[5,34]^。

LC-MS/MS因具有高灵敏度、高选择性等独特优势,已广泛应用于化学分析行业,尤其在残留分析领域。目前,国内外暂未见LC-MS/MS同时检测环境样品(土壤、沉积物和水)中ISO、MET和SAF残留的报道。基于此,本实验选取不同类型环境基质(土壤、沉积物和水),建立了一套环境样品中ISO、MET和SAF的固相萃取结合LC-MS/MS的残留检测分析方法,可为环境样品和环境行为试验样品中这3种除草剂残留的检测提供参考方法。

## 1 实验部分

### 1.1 仪器与设备

液相色谱-串联质谱联用仪(UPLC XEVO TQ-XS,美国Waters公司)、电子天平(AB104-S,梅特勒-托利多国际贸易上海有限公司;SE602F,奥豪斯常州仪器有限公司)、DHZ-DA恒温振荡器(江苏太仓实验设备厂)、R201旋转蒸发仪(上海申胜生物技术有限公司)、HH-4恒温水浴锅(江苏金坛市江南仪器厂)、SHZ-D(Ⅲ)循环水式真空泵(巩义市予华仪器有限责任公司)、57265固相萃取仪(美国Supelco公司)以及实验室其他常规仪器设备。

### 1.2 试剂与耗材

乙腈、甲醇(色谱纯,美国Fisher公司),乙腈(分析纯,上海凌峰化学试剂有限公司),甲酸(色谱纯,ROE SCIENTIFIC公司),饮用纯净水(广州屈臣氏食品饮料有限公司)。

ISO、MET和SAF标准品(纯度分别为99.5%、99.5%、98.3%,美国Chemservice公司)。

HLB小柱(500 mg/6 mL,美国Waters公司), ProElut C_18_小柱(500 mg/6 mL,北京迪马科技有限公司)。

### 1.3 标准溶液配制

用甲醇配制ISO、MET和SAF单标准储备液,然后用甲醇稀释标准储备液得到10 mg/L的混合标准工作液,再将混合标准工作液用甲醇稀释,得到0.0005、0.001、0.002、0.005、0.01和0.02 mg/L系列标准工作溶液。同时用土壤、沉积物和水空白基质配制相同浓度的基质标准溶液。

### 1.4 样品信息

土壤和沉积物性质见表1。实际水样的pH值为7.74,总有机碳含量(total organic carbon content, TOC)为0.738 mg/L。

**表 1:** 土壤和沉积物样品的性质

Sample	pH	Organic matter/ (g/kg)	Cation exchange content/ (mmol/kg)	Soil texture
Red soil	4.42	13.0	8.76	sandy loam soil
Paddy soil	5.97	28.5	17.7	sandy loam soil
Black soil	6.17	32.2	21.8	silt loam
Fluvo-aquic soil	8.72	9.5	8.68	sandy loam soil
Sediment 1	6.89	79.4	27.51	heavy loam soil
Sediment 2	7.15	9.6	8.49	light loam soil

### 1.5 样品前处理

#### 1.5.1 提取

土壤/沉积物:称取样品10 g(精确至0.01 g)于三角瓶中,再加入30 mL乙腈和15 mL水,振荡30 min后抽滤,滤液转移至装有8 g氯化钠的具塞量筒中,剧烈振摇约2 min,静置约2 h,吸取6 mL上清液,于40 ℃条件下浓缩至近干,用10 mL水复溶,超声后待净化。

水:将水样用滤纸过滤后移取10 mL于容器中,待净化。

#### 1.5.2 净化

土壤/沉积物:用5 mL乙腈和5 mL水预淋洗固相萃取小柱,将待净化液全部转移上柱,然后依次用5 mL 20%甲醇水溶液、5 mL 30%甲醇水溶液和5 mL 50%甲醇水溶液淋洗并弃去,最后用10 mL甲醇洗脱并收集,过0.22 μm滤膜后待进样。

水:移取10 mL水样上柱(固相萃取小柱先用5 mL乙腈和5 mL水预淋洗),再依次用5 mL 20%甲醇水溶液、5 mL 30%甲醇水溶液和5 mL 50%甲醇水溶液淋洗并弃去,最后用10 mL甲醇洗脱并收集,过0.22 μm滤膜后待进样。

### 1.6 样品检测条件

#### 1.6.1 液相色谱条件

色谱柱:Waters Acquity UPLC^®^ HSS C_18_色谱柱(100 mm×2.1 mm, 1.8 μm);流动相A:甲醇,流动相B: 0.1%(v/v)甲酸水溶液;柱温:40 ℃;流速:0.2 mL/min;进样量:1.0 μL。梯度洗脱程序:0~1.0 min, 60%A; 1.0~2.0 min, 60%A~90%A; 2.0~3.0 min, 90%A; 3.0~4.0 min, 90%A~60%A; 4.0~5.0 min, 60%A。

#### 1.6.2 质谱条件

离子源:电喷雾离子源(ESI);离子源温度:150 ℃;扫描方式:正离子多反应监测(MRM)模式;喷雾电压:3000 V;脱溶剂温度:400 ℃;脱溶剂气流量:800 L/h。其他质谱参数见表2。

**表 2:** 3种除草剂的保留时间和质谱参数

Compound	Retention time/min	Ion pairs (m/z)	Collision energies/eV
Isoxaflutole (ISO)	2.42	360.0/250.9^*^, 360.0/219.9	14, 40
Metazachlor (MET)	2.43	278.0/210.0, 278.0/134.1^*^	10, 22
Saflufenacil (SAF)	2.60	501.1/349.0, 501.1/198.0^*^	20, 38

* Quantitative ion.

## 2 结果与讨论

### 2.1 实验条件考察

#### 2.1.1 色谱和质谱条件确定

考察了Zorbax Eclipse XDB-C_8_(50 mm×2.1 mm, 1.8 μm)、DIKMA Endeavorsil C_18_(100 mm×2.1 mm, 1.8 μm)、BEH C_18_(100 mm×2.1 mm, 1.7 μm)、Xterra RP18(100 mm×2.1 mm, 3.5 μm)、BEH Hilic(50 mm×2.1 mm, 1.7 μm)和HSS C_18_(100 mm×2.1 mm, 1.8 μm)色谱柱对ISO、MET和SAF的保留和分离情况。由于Xterra RP18的填料粒径大,所有化合物在该柱上的响应很低,该柱不适合。所有化合物在BEH Hilic柱上0.6 min内出峰完毕,无保留,而且峰形也不对称,响应不理想。所有化合物在Zorbax Eclipse XDB-C_8_、BEH C_18_、Endeavorsil C_18_、HSS C_18_柱上均有较好的保留行为,但在HSS C_18_柱上响应最高,故选择了该分析色谱柱。

同时,考察了甲醇-水、甲醇-甲酸、甲醇-甲酸-醋酸铵和乙腈-甲酸-醋酸铵等流动相体系对化合物质谱响应值的影响。结果表明,流动相中酸的添加能明显增加化合物的质谱响应值,而醋酸铵的添加对化合物的质谱响应值影响不大,以甲醇为有机相比乙腈能获得更好的峰形。因此,流动相选择甲醇-甲酸水体系。

最后,考察了电离模式,并优化了锥孔电压和喷雾电压等质谱参数,确定了ISO、MET和SAF的母离子和子离子。MET和SAF适合电喷雾正离子采集模式(ESI^+^), ISO两种模式均可,但在ESI^+^模式下ISO的质谱响应值远高于其在ESI^-^模式下的质谱响应值。最终确定ISO、MET和SAF均采用ESI^+^模式检测。

综上,在优化的仪器条件下,3种除草剂的提取离子色谱图见图1。

**图 1:**
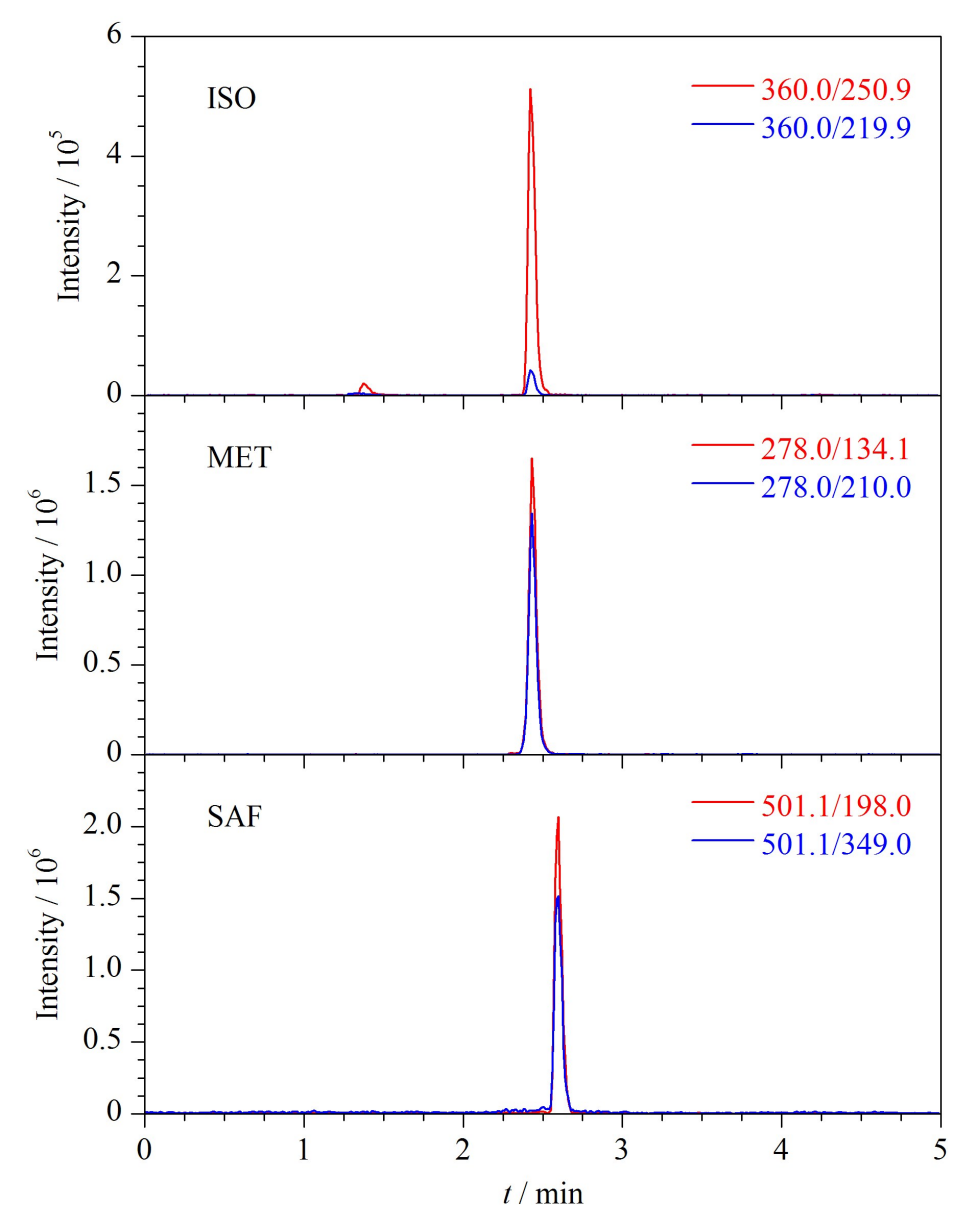
异噁唑草酮、吡唑草胺和苯嘧磺草胺(0.005 mg/L)的提取离子色谱图

#### 2.1.2 前处理条件确定

药物残留常用乙腈、甲醇、丙酮、乙酸乙酯和二氯甲烷等有机试剂为提取溶剂,由于二氯甲烷毒性较大,乙酸乙酯适用药物不多,丙酮提取干扰物较多且属于管控试剂,甲醇提取液不利于盐析,故本实验固态样品选择乙腈为提取试剂,液态样品则直接净化。

常用的净化方法有液-液萃取净化法^[31]^、分散固相萃取净化法^[35,36]^、固相萃取小柱净化法^[28,30,33]^、QuEChERS净化法^[23,24,26,27,29,34]^等,也有的不采取净化手段直接进行检测^[25,32,33]^。虽然QuEChERS净化法具有快速、简单、便宜等优点,但其净化效果有限,基质效应强,且存在污染仪器和影响外标法定量准确性等问题,通常需要购买价格较贵的同位素内标来校正^[37]^。样品不净化虽流程简单,但对仪器和外标法准确定量产生巨大挑战,无法应用于大量样品准确分析。

本实验选择了固相萃取小柱净化法,考察了ISO、MET和SAF在HLB和ProElut C_18_两款小柱上的保留情况。首先,用10 mL 20%乙腈水溶液复溶氮气吹干后的混合标准溶液,然后进行上样,并用含不同体积分数(30%、40%、50%、60%、80%)乙腈的水溶液依次淋洗固相萃取小柱,每种体积分数的淋洗体积均为3 mL,分别收集从上样开始的每种体积分数的流出液并测定其中药物浓度,分别计算各药物的回收率。结果显示:(1)过ProElut C_18_柱时,SAF在上样液中被部分淋出(占35.3%),保留较弱;ISO和MET分别在40%和50%乙腈水溶液中开始被淋出,保留较强。(2)在过HLB时,SAF在30%乙腈水溶液中被部分淋出(占31.6%), ISO和MET分别在40%和50%乙腈水溶液中开始被淋出。可见,3种除草剂在2种固相萃取柱上均有一定的保留,SAF在HLB柱上保留更强,但ProElut C_18_过柱速度更快,且价格优势明显,故选择ProElut C_18_柱进行后续试验。

前期采用乙腈-水体系淋洗ProElut C_18_柱时发现SAF在上样溶液中的淋出比例较高,说明采用乙腈-水体系淋洗能力较强,需更换为淋洗能力比其弱的甲醇-水体系进行淋洗优化。为了更好地去除样品共提取干扰物,淋洗优化时采用10 mL水复溶氮气吹干后的混合标准溶液,然后进行上样,并用含不同体积分数(20%、30%、50%、100%、100%)甲醇的水溶液依次淋洗固相萃取小柱,每种体积分数的淋洗体积均为5 mL。从图2可见,ISO、MET和SAF在上样溶液、20%和30%甲醇水淋出液中均未被淋出,在50%甲醇水淋出液中分别被淋出0、0.2%和0.5%,在第一段100%甲醇淋出液中分别淋出96.7%、98.0%和97.1%,在第二段100%甲醇淋出液中分别淋出3.3%、1.9%和2.4%。故最终确定过柱条件为:10 mL水溶液为上样溶液,依次用5 mL 20%甲醇水溶液、5 mL 30%甲醇水溶液和5 mL 50%甲醇水溶液淋洗去除干扰物,最后用10 mL甲醇洗脱目标化合物。

**图 2:**
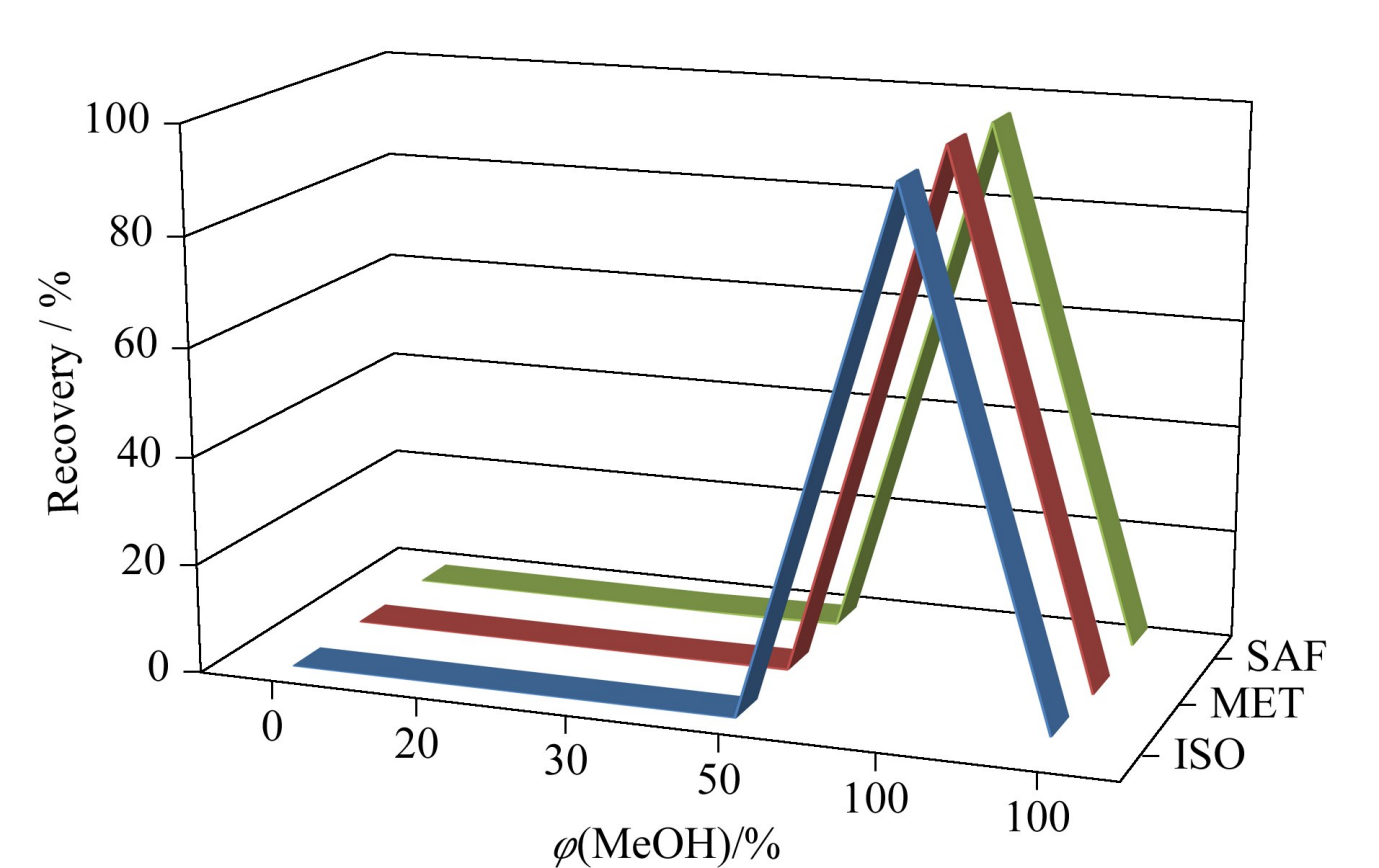
含不同体积分数甲醇的水溶液依次淋洗时异噁唑草酮、吡唑草胺和苯嘧磺草胺的回收率

### 2.2 方法学考察

#### 2.2.1 基质效应

环境样品主要包括土壤、沉积物、水和大气等,类型较多,组成成分各异,尤其是土壤和沉积物,基质比较复杂,净化不干净会造成较强基质效应,污染仪器,并影响准确定量。称取空白的4种土壤、2种沉积物和水样,按照1.5节和1.6节进行前处理和检测分析,确定其不含3种除草剂,再用空白样品溶液和甲醇溶剂配制0.0005~0.02 mg/L的基质标准溶液和溶剂标准溶液,将基质标准溶液和溶剂标准溶液在仪器上分析,以待测物的质量浓度为横坐标,峰面积为纵坐标绘制标准曲线,计算基质效应。基质效应计算见公式1。


(1) ME=(*A/B*-1)×100%

式中:*A*为基质匹配标准曲线的斜率;*B*为溶剂标准曲线的斜率。当基质效应绝对值<20%时,可用溶剂标准溶液进行定量;当基质效应绝对值≥20%时,需采用基质匹配标准溶液计算^[38,39]^。

3种除草剂在土壤、沉积物和水中的基质效应见图3。从图3可见,4种土壤、2种沉积物和水样中ISO的基质效应为-5.5%~13.6%; MET的基质效应为-10.1%~2.0%; SAF的基质效应为-5.7%~16.5%。3种除草剂的基质效应绝对值最大值为16.5%,均<20%,故不存在明显基质效应,表明净化效果较好,可以采用溶剂标准溶液定量。

**图 3:**
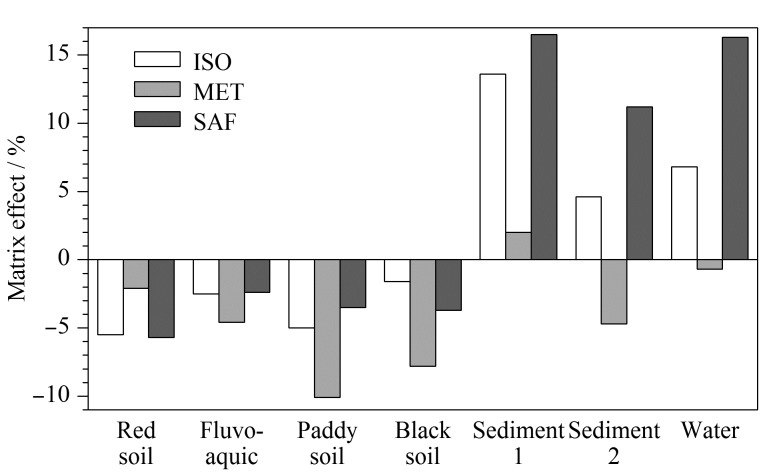
3种除草剂在土壤、沉积物和水中的基质效应

#### 2.2.2 线性关系、检出限和定量限

在0.0005~0.02 mg/L范围内,ISO、MET和SAF的线性关系均良好,*r*值分别为0.9972、0.9970和0.9961。根据空白样品的3倍信噪比确定方法的检出限,10倍信噪比确定方法的定量限,ISO、MET和SAF的检出限分别为0.05、0.01、0.02 μg/kg,定量限分别为0.2、0.05、0.05 μg/kg。

#### 2.2.3 回收率和精密度

为了确保方法的通用性,选取了不同的空白土壤、沉积物以及水样品进行了方法验证。在空白土壤、沉积物和水中分别添加0.005、0.1和2.0 mg/kg的ISO、MET和SAF混合标准溶液,每个水平平行6份,添加标准溶液后至少静置30 min,然后按照1.5节和1.6节方法进行前处理和仪器检测,平均回收率和相对标准偏差(RSD)结果见表3。3种除草剂在土壤中的平均回收率为81.8%~104.0%,RSD为1.2%~11.5%;在沉积物中的平均回收率为77.2%~103.4%,RSD为1.5%~12.8%;在水中的平均回收率为77.9%~107.1%,RSD为1.8%~6.0%。可见,本方法稳定、定量准确、实用性强,可用于土壤、沉积物和水样中3种除草剂残留量的检测。

**表 3:** 3种除草剂在土壤、沉积物和水中的加标回收率和RSD(*n*=6)

Matrix	Spiked level/ (mg/kg)	MET			ISO			SAF	
		Recovery/%	RSD/%		Recovery/%	RSD/%		Recovery/%	RSD/%
Red soil	0.005	88.9	3.1		94.7	5.5		97.5	6.5
	0.1	95.8	6.1		95.6	8.5		94.9	7.9
	2.0	91.7	3.8		88.8	5.7		81.8	5.9
Fluvo-aquic soil	0.005	90.6	4.1		96.6	4.5		96.0	8.3
	0.1	98.7	1.2		99.8	3.2		104.0	1.9
	2.0	96.5	7.7		98.5	8.1		92.7	11.5
Paddy soil	0.005	87.2	2.5		91.2	4.5		88.3	4.0
	0.1	94.3	0.4		101.9	1.6		95.5	2.5
	2.0	95.9	3.0		95.4	3.5		91.2	3.4
Black soil	0.005	88.5	3.4		93.4	3.2		94.9	3.6
	0.1	98.5	2.1		100.1	1.4		101.9	1.8
	2.0	91.6	4.3		89.8	2.9		82.9	3.2
Sediment 1	0.005	95.4	3.3		90.6	4.1		89.8	4.2
	0.1	102.4	4.0		91.9	2.5		97.8	1.5
	2.0	91.1	4.8		89.1	2.6		91.6	5.7
Sediment 2	0.005	90.6	1.7		77.2	8.2		93.4	2.8
	0.1	102.0	3.5		84.8	12.8		103.4	3.3
	2.0	101.2	4.4		88.7	5.4		102.6	7.6
Water	0.005	77.9	5.7		79.6	4.6		80.8	6.0
	0.1	91.3	1.9		92.1	3.9		93.8	3.0
	2.0	105.1	2.8		101.6	2.8		107.1	1.8

### 2.3 与已有方法比较

本方法与已有文献结果比较见表4,可以看出:1)文献报道均是关于这3种除草剂的单残留检测,而本方法可以同时检测样品中ISO、MET和SAF的残留量;2)本方法灵敏度高,定量限低,优于文献报道^[5,20⇓-22,30,32⇓-34]^;3)本方法基质效应不明显,明显优于文献报道^[5,30,33]^。

**表 4:** 本方法与已有方法比较

Ref.	Analyte	Pretreatment	Detection method	Matrix effects/%		Recoveries/%		LOQ/(mg/kg)
[5]	ISO	QuEChERS	LC-MS/MS (ESI^-^)	-28.78	-48.78	72.9	-116.5	0.005
[20]	ISO	SPE	HPLC		/	85	-104	0.1
[21]	ISO	SPE	HPLC		/	75	-105	0.1
[22]	MET	liquid-liquid distribution	HPLC		/	90.3	-99.0	0.1
[32]	MET	unpurified	GC		/	90.35	-106.24	0.05
[33]	MET	SPE (water)	LC-MS/MS (ESI^+^)	-94	-198		60	0.0002
		unpurified (sediment)					118	0.0001
[34]	MET	QuEChERS	LC-MS/MS (ESI^+^)	-7.3		86.5	-99.3	0.005
[30]	SAF	SPE	LC-MS/MS (ESI^+^)	-23.5	-21.5	88.9	-110.3	0.006
This method	ISO	SPE	LC-MS/MS (ESI^+^)	-5.5	-13.6	77.2	-101.9	0.0002
	MET			-10.1	-2.0	77.9	-105.1	0.00005
	SAF			-5.7	-16.5	80.8	-107.1	0.00005

### 2.4 方法应用

在浙江(萧山、台州、东阳和余杭)和黑龙江(佳木斯)的农田、林地、河流、湖泊采集了不同类型土壤、沉积物和水样样品,应用本方法同时检测样品中ISO、MET和SAF的残留量。由于这3种新型除草剂登记时间不长,目前应用还不是很广泛,所以,浙江和黑龙江收集的实际样品中均未检测到相关药物残留。

## 3 结论

本实验以4种不同类型土壤、2种沉积物和水为基质,开展了ISO、MET和SAF残留分析方法研究,对仪器检测条件和前处理条件进行了考察。结果表明:建立的残留分析方法操作简单,方法稳定,定量准确,实用性强,能满足环境样品中ISO、MET和SAF的痕量残留分析,可为这3种除草剂在环境样品中的残留污染监测和环境行为实验提供参考方法。
